# Supporting successful recruitment in a randomized control trial comparing clinic and home-based exercise among adults with multiple sclerosis

**DOI:** 10.1186/s40900-022-00366-6

**Published:** 2022-07-29

**Authors:** Tracy Flemming Tracy, Hui-Ju Young, Byron Lai, Beverly Layton, Dolly Stokes, Mark Fry, Tapan Mehta, Emily S. Riser, James Rimmer

**Affiliations:** 1 Tanner Foundation for Neurological Diseases, Birmingham, AL 35209 USA; 2 UAB/Lakeshore Research Collaborative, School of Health Professions, University of Alabama at Birmingham, Birmingham, AL 35233 USA; 3 Patient Author, Expert By Experience, Birmingham, AL USA; 4 Patient Author, Expert By Experience, Fairhope, AL USA; 5 Patient Author, Expert By Experience, Nashville, TN USA; 6 Department of Health Services Administration, School of Health Professions, University of Alabama at Birmingham, Birmingham, AL 35233 USA; 7 Tanner Center for Multiple Sclerosis, Birmingham, AL 35209 USA

**Keywords:** Multiple sclerosis, Telerehabilitation, Tele-exercise, Remote exercise, Complementary alternative medicine, Engagement, Stakeholder, Recruitment

## Abstract

**Background:**

The Tele- Exercise and Multiple Sclerosis (TEAMS) study, funded by the Patient Centered Outcome Research Institute (PCORI), is a pragmatic, cluster randomized controlled trial aimed at comparing the effectiveness of a 12-week complementary and alternative medicine (CAM) program for people with multiple sclerosis (MS) delivered by a therapist at a clinic and the same program initiated by the participant at home using a tablet and pre-recorded videos. The 20-session CAM program consists of yoga, Pilates and dual tasking exercises. The study aimed to enroll 820 participants with MS living in Alabama, Mississippi and Tennessee.

**Main Body:**

The information provided in this paper describes the strategies that led to the largest randomized controlled exercise trial ever conducted for people with multiple sclerosis. Specifically, the paper presents the result of incorporating stakeholder engagement, a novel participant recruitment method, to produce a successful recruitment outcome for a comparative effectiveness randomized controlled trial. This study used three tiers of engagement: panel members (9 members), clinical partners (88 occupational and physical therapists), and community organizations (6 non-profits).

**Conclusion:**

Engagement of the stakeholder panel, clinical partners and community organizations led to interest of over 1700 people with MS across three states in the Deep South (final enrollment was n = 837). The diversity of our stakeholder groups and their extensive reach into various communities were a critical aspect for achieving our target sample size. The recruitment numbers reflect the importance of involving multiple stakeholder groups at project inception, developing relationships over time, utilizing member strengths, and monitoring their engagement on a regular basis to ensure a meaningful experience for all involved.

*Trial registration*: NCT03117881. Registered 18 April 2017, https://clinicaltrials.gov/ct2/show/NCT03117881?term=tele+rehabilitation&cond=Multiple+Sclerosis&cntry=US&state=US%3AAL&draw=2&rank=1.

## Plain English language

The Tele Exercise and Multiple Sclerosis (TEAMS) study has been able to successfully screen over 1700 people with multiple sclerosis (MS) across three southern states (Alabama, Mississippi, Tenessee) largely due to the advice and input that the research team received from a stakeholder panel, clinic partners and community organizations. These groups met before the study was submitted for funding to the Patient-Centered Outcome Research Institute (PCORI), and was awarded in 2017. These engagement stages and framework established early in the study process were instrumental in generating strong enthusiasm for the study among various MS constituency groups. The feedback from our stakeholders, clinic partners, and community organizations led to the creation of a variety of recruitment methods (print material, email, social media, attendance at events, and health fairs) to connect with potential participants in a setting convenient within each location. In approximately 26 months, the study enrolled 837 participants with MS and baseline tested 759 individuals who participated in a rehabilitative exercise program at either a clinic site or in their home using a tablet that they were given (and kept) which included a set of preloaded videos. All milestones established by PCORI and research staff were met, leading to the largest exercise trail ever conducted with people with MS.

## Background

The recruitment and enrollment process is the most challenging phase of implementing a randomized controlled trial of exercise for people with disabilities, including people with multiple sclerosis (MS) [1]. A scoping review of published clinical trials for people with disabilities demonstrated that only 42% of those who are contacted agreed to participate in a research study actually join [1]. Thus, over half of the people who were asked to join a study either declined to participate or were excluded. This may be part of the reason that the average sample size of exercise trials for people with disabilities is 48 people per study or 21 people per study group (i.e., treatment and control group) [2]. Recruitment and enrollment delays can lead to extra costs for the study and potentially biased results, as well as underpowered and nonrepresentative samples that limit the generalizability and transferability of research findings [3, 4]. Another factor contributing to the hurdle of enrollment is the increased number of exercise trials offered to this population [5]. Exercise has become an important factor for disease prevention and symptom management for people with disabilities including MS leading researchers to attempt to validate frequency, duration, and type of exercises. Sample sizes in exercise trials have increased as well leading to a limited pool of potential participants [5].

To help provide insight into how to tackle the issue of recruitment and enrollment within exercise trials for people with disabilities, the information provided in this paper describes the strategies that led to the largest randomized controlled exercise trial ever conducted for people with MS. Specifically, the paper presents the result of incorporating stakeholder engagement, a novel participant recruitment method, to produce a successful recruitment outcome for a comparative effectiveness randomized controlled trial: *T*ele-*E*xercise *A*nd *M*ultiple *S*clerosis (TEAMS). The TEAMS study, funded by the Patient Centered Outcome Research Institute (PCORI), is a pragmatic, cluster randomized controlled trial aimed at comparing the effectiveness of a 12-week complementary and alternative medicine (CAM) program for people with multiple sclerosis (MS) delivered by a therapist at a clinic and the same program initiated by the participant at home using a tablet and pre-recorded videos [1]. The 20-session CAM program consists of yoga, Pilates and dual tasking exercises. The study aimed to enroll 820 participants with MS living in Alabama, Mississippi and Tennessee. The strategies utilized for stakeholder engagement in TEAMS was based on a description of engagement provided by PCORI. PCORI defines engagement as the “meaningful involvement of patients, caregivers, and other healthcare stakeholders throughout the entire research process, beginning from planning the study, to conducting the study and disseminating study results.” [1, 3] These principles framed the design of the TEAMS study, which contributed to the largest sample size observed in the extant published exercise literature in MS involving a study of CAM or exercise [1]. This commentary describes the stakeholder engagement strategy that led to the successful enrollment of 837 individuals with MS in the TEAMS study.

## Stakeholder engagement

The research staff used the definition of *engagement* outlined by PCORI and implemented it throughout the project by establishing three tiers of engagement: stakeholder panel, clinical partners, and community organizations. Detailed descriptions of each type of stakeholder group are provided below.

## Types of stakeholder

### Panel members

The stakeholder panel consisted of caregivers, exercise specialists, government officials, scientists, healthcare professionals, and non-profit employees (3 males, 6 females). Out of the nine panel members, five lived with MS.

### Clinical partners

**Fig. 1:**
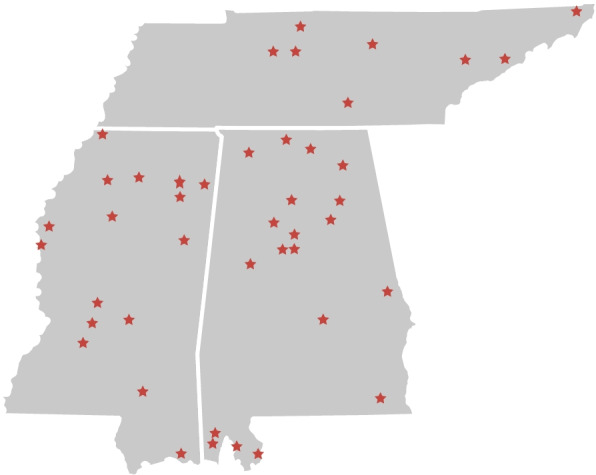
Clinic partners

The clinical partners consisted of five occupational therapists and 83 physical therapists and assistants at 43 clinics in three states: 19 in Alabama, 16 in Mississippi and 8 in Tennessee. Of these 43 clinic sites, 5 were considered small towns or urban clusters, 6 were considered urban clusters, and 32 were considered urban. There were a total of 88 therapists involved with the TEAMS study. Figure 1 highlights the geographic location of clinical partners within each state (Alabama, Mississippi, and Tennessee).

### Community organizations

**Fig. 2:**
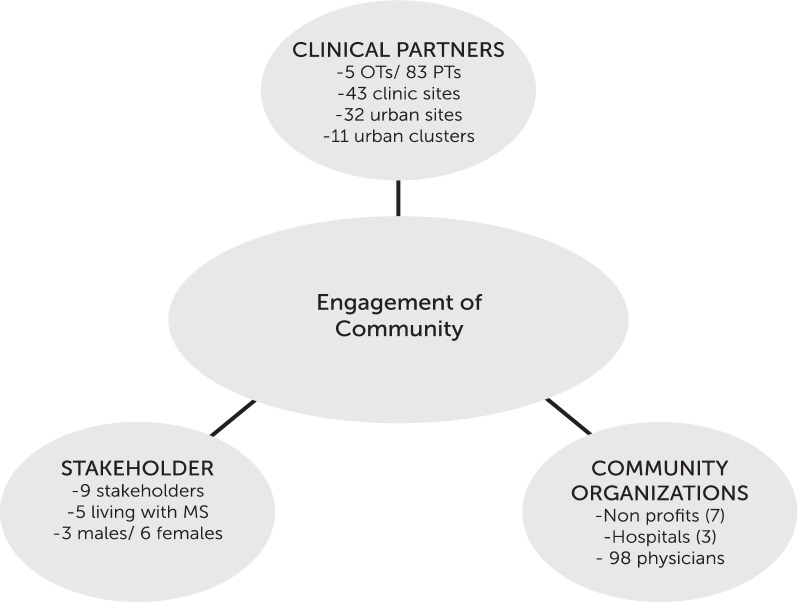
Member characteristics

The community organizations consisted of non-profits, hospital systems, neurology practices and other physician groups. The non-profit organizations include the National MS Society (NMSS), Multiple Sclerosis Foundation (MSF), MS Association of America (MSAA), MS Views and News (MSVN), iConquerMS, North American Research Committee on MS (NARCOMS), and the Consortium of MS Centers (CMSC). Hospital systems include Southeast Medical Center in Dothan, AL, Methodist Medical Center in Jackson, MS, and North Sunflower Medical Center in Ruleville, MS. Ninety eight neurology and primary care practicies in all three states participated in meetings with research staff. Study recruitment materials were provided to the practices who shared them with appropriate patients. Interested patients then obtained physician clearance to participate in the study (Fig. 2).

#### Engagement framework and stages

**Fig. 3:**
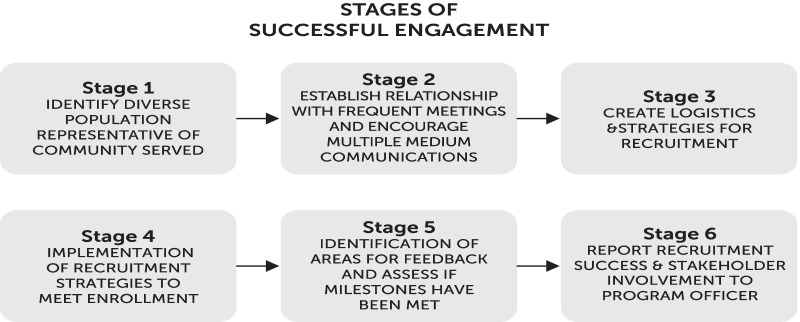
Stages of stakeholder engagement

Figure 3 illustrates the engagement strategies that were used in the TEAMS study to assist with recruitment. These engagement strategies were outlined in the research proposal and tracked monthly to ensure completion of study milestones and involvement of stakeholders at each stage. A total of six stages that were used throughout the four-year study in chronological order. In each stage, the input from the stakeholder groups laid the groundwork necessary for successful recruitment.

### Stage #1: Identify diverse population representative of community served

Prior to the grant submission, the stakeholder panel, the clinical partners and the community organizations were first contacted during the Letter of Intent process to gauge their level of interest. To determine invitations to the stakeholder panel, the research team met to identify individuals who had a connection to MS in Alabama, Mississippi and Tennessee. Close relationships with individuals developed over the past 26 years in the MS community from the PI and the Clinical Research Coordinator were leveraged to engage individuals to participate in this panel. Attention was paid to the background of each potential stakeholder in terms of their expertise, time availability, and interest in the study.

The clinical partners were identified based on their geographic location within each state. It was important to the success of the project to have clinic partners throughout each state to reach a diverse population from urban and rural areas and from clinics that were in close proximity to where participants lived for the onsite delivery of the clinical intervention arm of the study. Community organizations such as non-profit agencies and neurology practices were contacted based on previous relationships established prior to the TEAMS study, geographic location of the practice, and number of individuals with MS served by the organization or practice.

### Stage #2: Formalize relationships, set meeting schedule, and encourage multiple forms of communications

The stakeholder panel held its first in-person meeting prior to the submission of the proposal to PCORI to finalize the study design. During the meeting, primary outcomes of the study were discussed and finalized, which included fatigue, pain, physical activity, and quality of life. The stakeholder panel group interacted with the study team through four group meetings per year (two in person and two virtual meetings), individual conversations with panel members based on their area of expertise, and involvement in recruitment through attendance at local events to distribute study information. For the clinical partners, engagement strategies included in-person meetings, email, and phone correspondence for continued support, and coordination of their involvement of clinic partners at local events. In-person meetings with the clinical partners occurred at least twice per year for the past four years. Initially, these meetings were conducted to identify recruitment opportunities within their communities which varied from state to state. Later, these meetings were utilized to discuss retention strategies for participants enrolled in their clinics. Community organizations were engaged to assist with recruitment through events, social media, and self-help group meetings.

Information about regarding the study was shared on MSVN social media pages and other organizations such as the NMSS, iConquerMS, MSAA, and MSF were able to provide study information on their websites. The NMSS did a feature article in their magazine, Momentum, to promote remote exercises for the MS population. Members of the research team attended the CMSC and NMSS Annual Meetings, NMSS walk events, and visited each MS self-help group in the states of Alabama (6 groups), Mississippi (7 groups), and Tennessee (12 groups) at least once during the study. When available and in the area visited, a stakeholder panel member or a clinical partner would attend the self-help group meeting, walk event, and annual meetings with research staff. All these engagement opportunities had a positive impact within and across states.

### Stage #3: Create logistics and strategies for recruitment

**Fig. 4:**
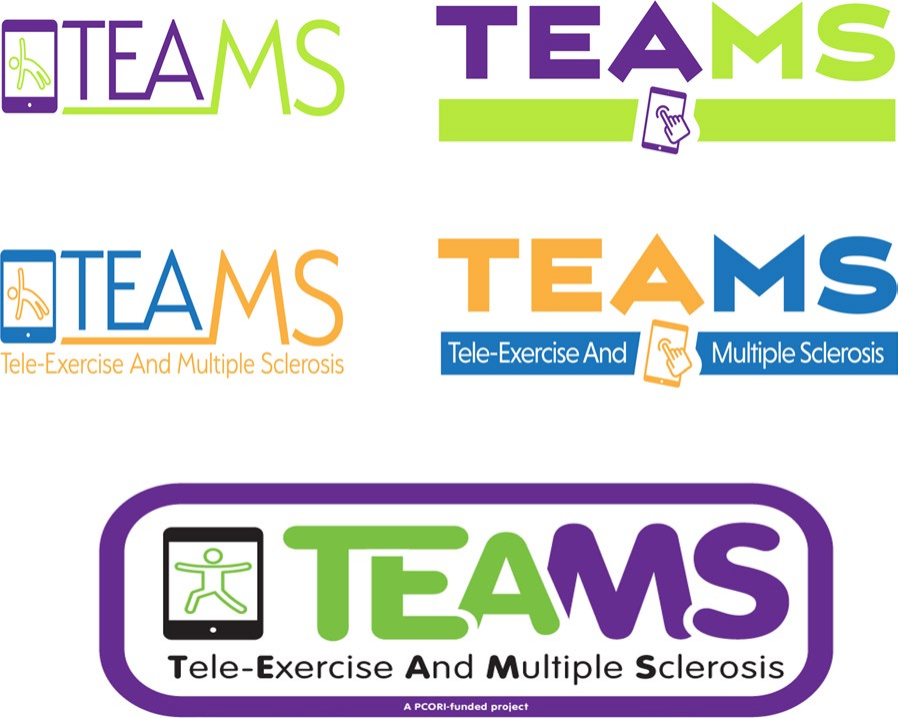
TEAMS logo iterations

The sample size target of the study, 820 participants, was presented to the stakeholder panel during the first meeting. A recruitment plan was formulated specific to each of the three states. In the first year of the study, stakeholders assisted with the development of an expansive recruitment strategy that varied from state to state and considered both urban and rural areas in each state. Each set of stakeholders was helpful in identifying local events, active self-help groups, and practices serving people with MS. Dissemination and communication strategies varied by rural versus urban areas in each state. In some rural areas, churches were identified as key gathering places for spreading the word about the study. In contrast, in urban areas, coffee shops were a bigger draw for recruitment events. To ensure good name recognition for the TEAMS study, a logo was developed by the stakeholder panel and a design vetted for the creation of a brochure and flyer. Figure 4 represents the iterations in the logo being developed with the last logo used on all print material and exercise equipment provided to the participants. These flyers were utilized in the areas identified by the stakeholders either in printed versions in physician waiting rooms, through mailings to homes, and/or social media posts made by organization partners.

### Stage #4: Implementation of recruitment strategies to meet enrollment

After the flyer design was approved by the stakeholder panel, the first recruitment push involved hand writing addresses on brochures that were sent to a mailing list of over 4000 individuals with MS,in addition to in-person delivery of flyers to approximately 55 physician offices. A study website was created, and usability tested by the stakeholder panel to ensure that online enrollment into the study would be as seamless and effortless as possible. Feedback during this stage did not identify any problems and the website was able to be launched with content and photos used in the flyer and brochure design, which was decided by the panel. During the study, the panel assisted with identifying important recruitment events that would be crucial areas for recruitment and determined what information needed to be provided at these events. Later in the study, during the last recruitment push in year three, to continue to meet recruitment milestones the research staff asked the stakeholder panel to develop a letter that was sent out to physician offices to encourage referrals into the study to achieve its target sample size.

The 88 clinicians across Alabama, Mississippi, and Tennessee who were involved in the delivery of the TEAMS intervention study were able to assist with recruitment in three ways: (a) use of their Electronic Medical Records to identify additional participants with MS; (b) identify physicians treating patients with MS within their community; and (c) attend local events to distribute study information. The clinicians also connected TEAMS research staff with local newspapers and magazines to feature information regarding enrollment in the TEAMS study.

The MS organizations (NMSS, MSF, MSVN, MSAA, and CMSC) facilitated recruitment opportunities for TEAMS research staff to speak at local events, obtain booth space, and assist with social media outreach. Additionally, the NMSS assisted with identifying the number of potential participants in each state, provided names of physicians treating patients with MS by geographic area, and provided access to self-help group leaders for community outreach. NARCOMS and iConquer MS offered mailing and recruitment assistance for specific areas. Neurologists and other physician groups were also engaged during this process to outline ways for recruitment within their practice, determine timeliness of communication between research staff and physician offices for medical clearance and study progress, and vet any concerns of office staff and participants.

### Stage #5: Identification of areas for feedback and assess if milestones have been met

Each stakeholder panel meeting had a formal agenda that included study updates, recruitment numbers, preliminary data (e.g., sample demographics), findings (when available), and an open discussion on any issues or items that needed advisement from the stakeholders.. This draft agenda was developed by the researchers and sent out to the stakeholders for additional items and/or discussion points and final approval. During the meeting, open ended questions were posed to the panel for suggestions and input on study design and strategies for enhancing recruitment. Clinicians were offered an opportunity to provide feedback in an online survey to gauge their engagement in the study. Clinicians also reported any updates and efforts in recruiting via email or at the bi-yearly in-person meetings. During the annual meeting of our stakeholder organizations, events and self-help group meetings and updates were provided on recruitment efforts and suggestions were requested from attendees to identify best practices for community outreach.

### Stage #6: Report recruitment progress and stakeholder involvement to program officer

Recruitment progress was cataloged monthly and reported to our funding agency, PCORI, through a monthly report, a monthly phone call, and an Interim Progress Report (IPR) submitted every six months. Recruitment numbers reported through a Consort diagram outlined total enrolled, baseline tested, follow-up tested, and lost to follow up. The monthly phone call with PCORI involved the PI, coordinators, and program officer. Discussions involved updates on milestones completion and any new challenges. During the monthly calls updates on stakeholder involvement were provided to the program officer. Detailed descriptions were reported monthly through the IPR in narrative form delineating engagement of each stakeholder group for the previous six months. Each of these reports was also presented at stakeholder panel meetings, through email communication with clinicians and physician offices, and in presentations for self-help groups and local events. The program officer also attended stakeholder meetings virtually when available.

## Engagement and recruitment outcomes

Engagement of the stakeholder panel, clinical partners, and organizations at study inception and throughout the scope of work provided several opportunities for improving the quality of the research through enhanced credibility, generalizability, and feasibility of implementation. Opportunities presented themselves through co production of the intervention, videos, and study materials, and edits and modifications were made by the stakeholders, clinicians, and organizations, in addition to their confirmation of study content, protocols, and recruitment processes.

Table 1 outlines the Engagement Details of each stakeholder group involved in the TEAMS study.

**Table 1:** Engagement details

Year	Stakeholder	Clinical partners	Organizations
2016	Identified clinic sites, primary and secondary outcomes, and outcome measures Logo and Intervention development Review Informed Consent Determined Interactive voice response content	Letter of support and FFS Agreements obtained from Drayer Physical Therapy, Montgomery East Physical Therapy, Gulf Coast Therapy, Therapy Achievements, Southeast Alabama Medical Center, Tanner Center, and Methodist Rehabilitation Center Clinics worked to identify recruitment strategies, transportation, internet services storage of equipment and files specific to each clinic site and community served	Letters of Support- MS Coalition: Consortium of MS Centers (CMSC), National MS Society (NMSS), Multiple Sclerosis Foundation (MSF), MS Association of America (MSAA), and MS Views and News (MSV&N)
2017	Determined articles for tablet app, and tablet app videos format Reviewed and modified MOP and study brochure IVR call frequency. length and voice determined Added Tennessee stakeholder and clinics to the study	Six Drayer Physical therapy clinic sites added in Tennessee and Encore Rehabilitation Pilot testing at Montgomery East Physical Therapy and Tanner Center Best communication with Clinical Research Coordinator established for each clinician through Box, email, and/or phone Communication with other coordinators through Box	Recruitment: NMSS, MSF, MSAA, MSV&N, North American Registry of MS (NARCOMS) and iConquerMS Intervention, Training Manual and Outcome measures: CMSC Abstract presentations at American Congress on Rehabilitation Medicine
2018	Pilot testing with stakeholders for testing protocols, IVR, and tablet app Established timeframe for screening to testing and for any pause in intervention Designed postcard for retention and survey for participants and therapists	Added River City Rehabilitation and North Sunflower Medical Center: training and testing completed Clinicians continue to recruit for the study through attendance at events and handing out brochures at the clinics Timing of intervention and testing adjusted based on feedback Protocols designed to improve communication, equipment and gift card inventory for each clinic site	Neurology practices in Alabama, Mississippi and Tennessee through meetings and providing study information to patients NMSS Walk Events and self-help group meetings MSAA and MSV&N speaking opportunities and booths at events
2019	Revamped IVR to improve quality and lessen call length Established Lost to contact protocol Second postcard designed for retention Identification of areas for recruitment: events, letters to physicians, self-help group meetings	Clinicians assisting with initial scheduling, lost to contact and withdraw protocol Equipment and gift card inventory return coordinated for clinics finished with the study for redistribution at other clinics Clinicians assisting obtaining missing data in collaboration with the Clinical Research Coordinator	Recruitment continued through NMSS and MSV&N events and meetings Abstracts presented at European Committee for Treatment and Research in MS (ECTRIMS) and presentation at PCORI Annual Meeting on Engagement Article in Momentum NMSS magazine
2020	Email developed to update therapists on study progress for encouragement Dissemination ideas discussed: publications, presentations at meetings, participant/caregiver events Feedback provided regarding change of study to all Tele platform	Consent for therapist survey and interview sent to 42 clinicians 14 surveys completed 16 therapists completing training for Tele Assessment and 6 therapists participating in remote DirectCAM Inter-Rater reliability performed with therapists at the Tanner Center	Abstracts presented at CMSC and American Academy of Neurology (AAN) Presentation scheduled at Institute for Patient- Family Centered Care (IPFCC) on engagement ACRM provided information on Tele rehabilitation during COVID-19 via virtual meetings

**Fig. 5:**
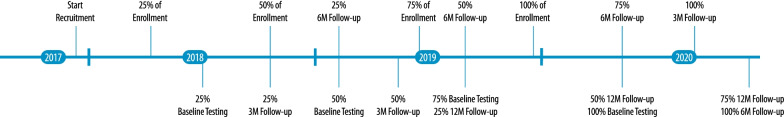
Achieved milestones of enrollment and follow-up testing timeline

The impact and influence of stakeholder engagement had a substantial effect on screening over 1700 individuals with MS across Alabama, Mississippi and Tennessee through mailings, study website, social media, events, distribution of recruitment materials at clinics and physician offices and presentations at self-help groups. Nineteen Alabama clinics were able to enroll 523 individuals, the sixteen Mississippi clinics enrolled 196 participants and the eight Tennessee clinics were able to enroll 118 participants. The average number obtained by the 43 clinics was 19 participants. Participant recruitment by clinic ranged from four participants at a small-town urban cluster clinic to 56 participants at an urban clinic. When querying our database on how participants found out about the study, the highest response was through a study flyer, followed by word of mouth, and through clinic staff. Although we are unable to quantify the influence of the early strategies developed by our stakeholders on participant enrollment, the stakeholder group likely led to successful engagement of clinical partners and various organizations increasing our successful recruitment efforts in each state (Fig. 5).

## Discussion

The success of the described stakeholder engagement strategies is reflected in the recruitment numbers and the ability to meet enrollment milestones. The diversity of our stakeholder groups and their extensive reach into various communities were a critical aspect for achieving our target sample size (n = 820). To our knowledge, this is the largest exercise rehabilitation trial ever conducted on people with MS. It should also be noted that recruitment in the Deep South presented its own challenges. Many blacks are skeptical of participating in research and there has been a history of mistrust between researchers and the black community [6]. The backgrounds among the stakeholders involved in this randomized controlled trial helped to promote the study throughout the Deep South. Engagement activities were tailored to the stakeholder panel, clinic partners, and community organizations that were aligned with their knowledge and affiliations, helping to reach people with MS within their communities. This, along with the variety of communication methods implemented, allowed for sustained involvement of the stakeholders throughout the project despite a change in working conditions due to the COVID-19 pandemic.

Successful engagement, however, is not without its challenges. Due to the length of the TEAMS study (4 years), one valued member of our stakeholder panel experienced health conditions in year three and one had to deal with health issues of a family member that did not allow them to continue serving on the panel in year four. In addition to the loss of two stakeholder panel members, some of our clinic partners and non-profit organizations experienced furloughs or job cuts due to the pandemic and were no longer able to contribute to the study. Though the loss of these members was difficult because of the strong relationships that were developed, the recruitment of participants was not significantly impacted because of the involvement of the remaining stakeholders and organizations.

A limitation of the present study is that we were unable to quantify the influence of various strategies (e.g., stakeholder changes to recruitment procedures) on recruitment and enrollment because it was not the intended purpose of the study design. However, after achieving such a large sample size in a population of people with MS, we felt this retrospective commentary would be insightful to other researchers interested in our stages of engagement and to future PCORI-funded entities who need more awareness on who to strengthen their stakeholder engagement. Our ability to successfully enroll over 800 people with MS into one of the largest exercise RCTS (if not the largest), is a good indication that our extensive engagement methods by multiple stakeholders, clinics and organizations was a critical component for achieving a very large sample size in a low incidence population. For researchers interested in prospectively examining stakeholder engagement as a unique research question (vs. retrospectively as part of a large RCT), future research could include process evaluation of stakeholder contributions or a SWAT (study within a trial) to examine how recruitment with or without involvement would provide information needed to support its potential effect. Also, understanding how stakeholder engagement could be useful to retention of study participants would be a strong contribution of these kinds of studies in understanding the magnitude of importance in engaging various stakeholders.

## Conclusions

Engagement of the stakeholder panel, clinical partners and community organizations led to successful screening of over 1700 people with MS across three states in the Deep South (final enrollment was n = 837). The recruitment numbers reflect the importance of involving multiple stakeholder groups at project development, maintaining relationships over time, utilizing member strengths, and monitoring their engagement on a regular basis to ensure a meaningful experience for all involved. Building trust among existing community systems served as a key element of success in this research project.

## Data Availability

All data that will be used or analyzed in the study will be supplied upon reasonable request.

## Abbreviations

MS: Multiple sclerosis
PCORI: Patient Centered Outcome Research Institute
CAM: Complimentary Alternative Medicine
NMSS: National Multiple Sclerosis Society
MSF: Multiple Sclerosis Foundation
MSAA: Multiple Sclerosis Association of America
MSVN: MS Views and News
NARCOMS: North American Registry Committee on MS
CMSC: Consortium of MS Centers
